# Predictors of Escalation of Lipid-Lowering Therapy with Subanalysis of the Influence of Lipoprotein (a) on the Decision-Making Process

**DOI:** 10.3390/diseases14010008

**Published:** 2025-12-27

**Authors:** Paweł Muszyński, Kinga Natalia Dudzińska, Marlena Święcicka, Wiktoria Grądzka-Matys, Małgorzata Chlabicz, Dominika Musiałowska, Joanna Kruszyńska, Piotr Kazberuk, Urszula Bajda, Anna Tomaszuk-Kazberuk

**Affiliations:** 1 Department of Cardiology, Lipidology and Internal Diseases, Medical University of Bialystok, Żurawia 14, 15-569 Bialystok, Poland; 2 Department of Invasive Cardiology, Medical University of Bialystok, M. Skłodowskiej-Curie 24A, 15-276 Bialystok, Poland

**Keywords:** pharmacotherapy, lipid disorders, lipid-lowering therapies

## Abstract

**Background/Objectives:** Cardiovascular diseases are the leading cause of death worldwide. The preventive efforts to reduce the burden are crucial. Primary causes of cardiovascular diseases include lipid disorders. The variety of available medications influences cardiovascular risk and allows for improvement. However, discontinuation or infrequent initiation of lipid-lowering therapies remains a problem. This study aimed to investigate predictors of lipid-lowering therapy escalation. **Methods:** 431 patients with known concentrations of Lipoprotein (a) (Lp (a)) acquired as part of routine cardiovascular risk assessment from the HELPE-R registry, hospitalised in the University Clinical Hospital in Białystok were included in this study. Escalation of treatment was defined as the initiation of any form of lowering therapy or an increase in the potency or dose of statins. The analysis of the influence of various factors on the decision about escalation was performed. **Results:** The median age was 69.00 years. The escalation of therapy occurred in 48.49% of patients. Not reaching the LDL-C goal was the strongest predictor of escalation (OR: 9.177). The other factors increasing the probability of escalation included acute coronary syndrome (OR: 3.913), prediabetes (OR: 2.372), chronic coronary syndrome (OR: 2.217), dyslipidemia (OR: 2.354), hypertension (OR: 1.734), carotid artery stenosis (OR: 1.625), and obesity (OR: 1.543). There was no effect of past MI and stroke on the escalation of lipid profile. Lp (a) did not affect the escalation. **Conclusions:** The decision about escalation of lipid-lowering therapy is mainly influenced by classical risk factors and established atherosclerotic disease. Lp (a) did not affect the escalation, despite growing interest among medical practitioners.

## 1. Introduction

Cardiovascular diseases (ischaemic heart disease and stroke) remain the leading causes of death worldwide, and lipid disorders are a significant risk factor for the development of atherosclerosis and its complications [1,2,3]. Improvement in the lipid profile is achieved through lifestyle modifications (low-cholesterol diet, increased physical activity, weight reduction, smoking cessation) and the use of medications with proven efficacy (statins as first-line drugs, ezetimibe, PCSK9 inhibitors, bempedoic acid, fibrates—mainly for hypertriglyceridemia—and omega-3 fatty acids) [3,4].

Decisions regarding escalation of lipid-lowering therapy are based on clinical factors associated with cardiovascular risk. The cardiovascular risk assessment is based on established comorbidities or risk stratified by risk estimation in apparently healthy people, using the SCORE2 and SCORE2-OP algorithms. However, the number of factors included in these algorithms is limited to age, sex, systolic blood pressure, non-HDL-C, smoking status, and global risk category for the patients’ country of origin for [3,5]. Thus, the “true” risk could be underestimated.

The number of known risk factors is constantly increasing, and this is connected with extensive preventive measures, but still only 19% of patients achieve the LDL-C goal [4,6]. The summary of factors influencing the cardiovascular risk mentioned in the Guidelines and with established potential are presented in Table 1. Each of these should be included in the assessment and should encourage escalation of lipid-lowering therapy.

The goal of the treatment for a patient with a very high risk is to lower LDL-C levels to <55 mg/dL or by 50% from baseline. Within this group, we can distinguish a subgroup of patients at extremely high cardiovascular risk, with a target LDL-C of <40 mg/dL—patients with ASCVD who experience recurrent vascular events while taking maximally tolerated statin-based therapy, and patients with polyvascular arterial disease [4]. These patients require intensive treatment, often in form of combine therapy to achieve reduction of LDL-C [7]. The benefit is amplified with every reduction in LDL-C, even in patients with already very low LDL-C (“the lower is better” concept) [8,9,10]. Thus, decision about the escalation should be liberal and should include a holistic assessment of the patients’ risk. Currently, LDL-C concentration plays the main role in therapeutic decision-making, followed by non-HDL-C, but in recent years, increasing attention has been given to Lipoprotein (a) (Lp (a)). Lp (a) is a lipoprotein structurally similar to LDL but with an additional apolipoprotein (a). High Lp (a) levels increase total cardiovascular risk, and in individuals with borderline lipid profiles, prompt more intensive treatment. In studies and guidelines, Lp (a) is used for recalibrating cardiovascular event risk, meaning a patient with high Lp (a) may be reclassified into a higher risk category [4].

The current study aimed to assess the real-life impact of comorbidities and known risk factors of cardiovascular diseases on the decision about escalation of lipid-lowering therapy. The secondary goal was to investigate the role of the Lp (a) in the decision process in patients with different LDL-C levels.

## 2. Materials and Methods

The analysis was performed retrospectively, using data collected by the Department of Cardiology, Lipidology and Internal Diseases, Medical University of Bialystok, Poland, from December 2023 to January 2025 as part of the HELPE-R (Heading towards Exploration of Lp (a) Proarrhythmic Effect Registry). From the initial 1538 patients hospitalised at the University Clinical Hospital in Białystok for whom the Lp (a) concentration was recorded (inclusion criteria), 431 were included in the study. The exclusion criteria included a current lack of data regarding the lipid profile, comorbidities, and treatment at admission and discharge. The data collection was based on the medical documentation; no additional procedures/tests were performed. The Lp (a) measurement was a part of the routine diagnostic process. The analysis focused on determining the factors influencing the decision to escalate lipid-lowering therapy. The analysed factors included comorbidities, sex, lipid profile, and Lp (a). The escalation of treatment was defined as the initiation of any form of lowering therapy, increasing the potency or dose of statins. The risk categories and definitions were based on recent 2025 ESC update [11]. The current study is a subanalysis of early results from the outgoing registry, with missing data continuously being filled.

### 2.1. Statistical Analysis

Continuous variables are shown as median ± interquartile range (IQR). Categorical variables are presented as percentages (number of patients). The normal distribution was assessed using the Kolmogorov–Smirnov test. Statistical tests used for analysis included Student’s *t*-test for parametric continuous variables, the Mann–Whitney U-test for non-parametric continuous categorical variables, and the Chi-squared test and odds ratio Altman calculation for categorical variables. The statistics were performed using Statistica 13. Univariate logistic regression was used for assessment of influence of various factors on the decision about escalation of lipid lowering therapy. The multivariable logistic regression was used for assessment differences in impact of lipid profile between specific groups of patients according to LDL-C. The logistic regression was executed using Stata 18.0. Logistic regression coefficients present the outcome for a one-unit increase in the predictor variable. *p*-value ≤ 0.05 was considered significant. The pairwise deletion approach was used to handle missing data.

### 2.2. Ethics

This study was conducted in accordance with the Declaration of Helsinki and was approved by the Institutional Review Board of the Medical University of Bialystok on 19 December 2024 (APK.002.537.2024). Informed consent was obtained from all subjects involved in this study.

## 3. Results

In total, 431 patients (median age of 69 ± 19 years; 45.01% men) were included in the study. The lipid-lowering therapy was escalated in 48.49% (*n* = 209). The clinical and selected biochemical data of the study group were presented in Table 1.

There was no statistically significant difference in escalation of the therapy regarding age, sex, diabetes mellitus (DM), chronic kidney disease (CKD), peripheral artery disease (PAD), or heart failure (HF).

Patients, in whom therapy was escalated, were statistically significantly more often diagnosed with prediabetes (11% vs. 4,95%; *p* = 0.023), obesity (36.36% vs. 27%; *p* = 0.038), hypertension (80.38% vs. 70.27%; *p* = 0.016), dyslipidemia (82.78% vs. 67.12%; *p* < 0.001), chronic coronary syndrome (CCS) (37.32% vs. 21.17%; *p* < 0.001), and acute coronary syndrome (ACS) (6.70% vs. 1.80%; *p* = 0.018).

The occurrence of premature cardiovascular disease, family history of cardiovascular diseases and age at the first episode were similar in both groups.

**Figure 1:**
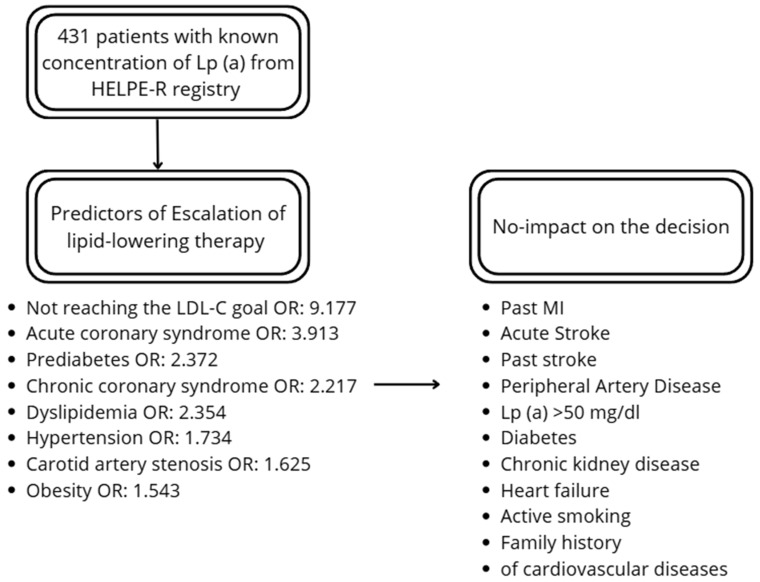
Summary of the predictors and factors not-influencing the decision.

The factors increasing the probability of escalation of the therapy included chronic coronary syndrome (OR: 2.217), acute coronary syndrome (OR: 3.913), carotid artery stenosis (OR: 1.625), hypertension (OR: 1.734), dyslipidemia (OR: 2.354), prediabetes (OR: 2.372), and obesity (OR: 1.543). There was no effect of past MI and stroke on the escalation. From the lipid profile, total cholesterol (TC), LDL-c and non-HDL-C had a significant impact on the decision. Lp (a) did not affect the escalation. The distance from LDL-C goal was higher among patients undergoing therapy escalation. Additionally, not reaching the LDL-C goal increased probability of escalation (OR: 9.177). Details are presented in Table 2 and Figure 1.

**Figure 2:**
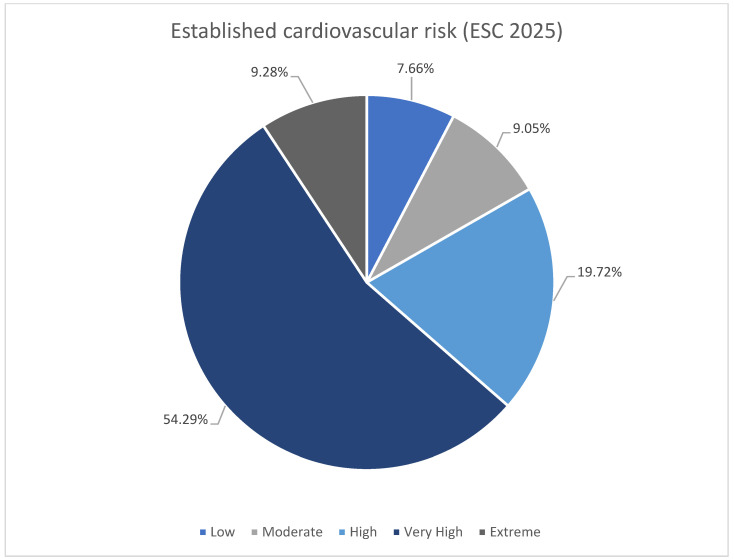
Summary of established cardiovascular risk (ESC 2025 [11]).

**Figure 3:**
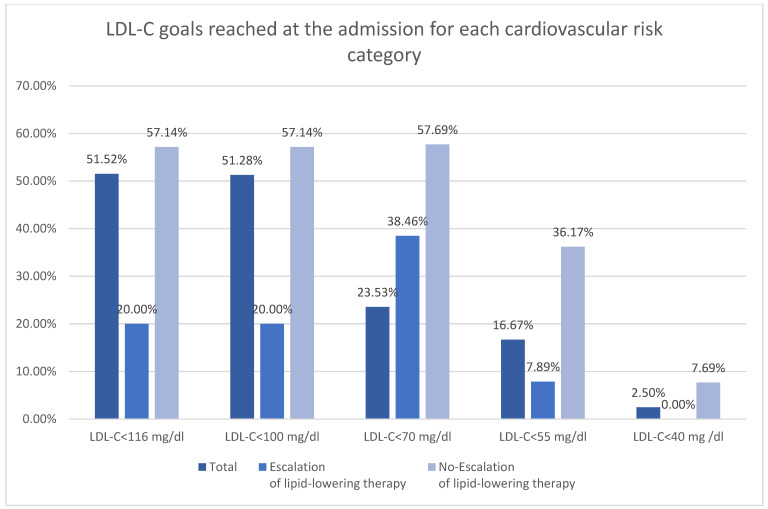
LDL-C goals reached at the admission for each cardiovascular risk category.

The subdivision of the patients according to ESC 2025 cardiovascular risk definitions was shown in Table 3 [11]. Majority of the patients were at very high risk in both groups (Figure 2). There was higher rate of low and moderate risk patient in no-escalation group. There was no difference in occurrence of high risk, however both very high risk and extreme risk was more prevalent among patient undergoing escalation. The LDL-C goals were more frequently reached among patients with low-moderate risk (Figure 3). The majority of patients with high, very high, and extreme risk were not within LDL-C goals (Table 3).

In patients with LDL-C above 100 mg/dL, there was no significant impact of any part of the lipid profile on the escalation of lipid-lowering therapy. When LDL-C was > 70 mg/dL, the increase in TC, LDL-C, triglycerides (TG), and non-HDL-C caused a higher probability of escalation. In patients with LDL-C > 55 mg/dL and not-within target goal, TC, LDL-C, and non-HDL-C influence lipid-lowering therapy (Table 4).

The prescription of statins and ezetimibe increased at discharge. There was no significant difference in the prescription of fibrates and PCSK-9 inhibitors (Table 5).

Patients within LDL-C goals more often received statin and ezetimibe at admission. Rosuvastatin was more often received by patients within the goals (Table 6).

The prescription of statin, ezetimibe, and PCSK-9i increased with growth of cardiovascular risk. The rosuvastatin was most prescribed statin, with increase in usage among patients with very high and extreme cardiovascular risk. The low-to-moderate intensity statins were similarly prescribed in every risk category. However, the prescription of high-intensity statins increased with higher cardiovascular risk. Dual and triple therapy were most frequently prescribed for patients with very high and extreme risk (Table 7).

Of patients undergoing lipid-lowering therapy escalations, 39.71% had statin initiation, 26.32% had an increased dose, 12.92% had ezetimibe invitations, and 1.91% PCSK-9i. Only 5.26% received up-front, combined lipid therapy. Statin initiation and increase of statin dose were more frequent among patients with distance to LDL-C goal < 50 mg/dL. Patients with distance to LDL-C goal ≥ 50 mg/dL more often received up-front combined lipid therapy (statin and ezetimibe). There was also a trend towards using PCSK-9i in patients with distance ≥50 mg/dL (*p* = 0.073) (Table 8).

## 4. Discussion

### 4.1. Decision-Making Process in Light of Current Guidelines

Clinical studies indicate that despite modern lipid-lowering therapies and studies confirming their effectiveness, a significant proportion of patients at cardiovascular risk do not achieve target LDL values [6,12].

The optimization of lipid-lowering therapy remains a cornerstone of both primary and secondary cardiovascular disease prevention. Contemporary Polish guidance aligned with recent European Society of Cardiology recommendations emphasizes more intensive LDL-C lowering in patients at high and very-high cardiovascular risk and advocates early combination therapy when monotherapy does not achieve target concentrations. This guidance explicitly recommend stratified LDL-C targets and a stepwise intensification of therapy, including escalation to high-intensity statins, addition of ezetimibe, and consideration of PCSK9 inhibitors for patients who remain above the target despite optimal statin-based therapy [3,4,5].

To systematize treatment escalation methods that will serve clinical practice, broader issues should be considered. For years, cardiovascular disease (CVD) has been the leading cause of death worldwide, especially ischemic heart disease (IHD) and stroke. Therefore, such work will constitutes a valuable resource for prevention in patients at high cardiovascular risk [12]. It is crucial to identify factors associated with escalation of lipid-lowering therapy (LLT), because these could indicate potential targets for education among medical practitioners. Risk factors not included in the decision-making process can increase the cardiovascular risk indicated by classical risk estimation. Thus, education can increase awareness regarding “true” risk and increase lifestyle intervention and prescription of medication with proven cardiovascular benefit.

Despite classical risk factors, the guidelines suggest initiation of lipid-lowering therapy in patients with chronic kidney disease, diabetes mellitus, and metabolic syndrome [3,13,14,15]. Many factors have been added as a risk modifier for consideration beyond the risk estimation based on the SCORE2 and SCORE2-OP algorithms. The most important of these include systemic inflammation and elevated Lp (a). Preventive measures based on proper risk category establishment and applying all available measurements including lipid-lowering therapies to reduce the risk burden [3,4,5]. Currently, lipid-lowering therapy plays crucial role in management of elevated Lp (a). AI machine learning models established and suggested by EAS have been implemented in a recent update of the guidelines, allowing prediction of potential benefit from lipid-lowering therapy in that group of patients. Available treatment includes life-style modifications, lipid-lowering therapy including PCSK-9i and lipid apheresis [16]. Outgoing trials could potentially allow implantation of Lp (a)-specific treatment (obicetrapib, pelacarsen, olpasiran, zerlasiran, lepodisiran, and muvalaplin) [17].

### 4.2. Search for Factors Influencing the Decision Regarding the Escalation

Studies on factors influencing decisions to initiate lipid-lowering therapies are limited. However, they can provide information on the implementation of guidelines in clinical practice and allow assessment of what improvements can be made. Similar studies have been performed in patients with diabetes mellitus [18,19]. A recent post hoc analysis of the SANTORINI study, including 9559 patients, showed that the majority of patients were not within LDL-C goal (74.0%) and that only 19% of patients underwent escalation of lipid-lowering therapy [6]. In our study, the proportion of patients who did not reach the LDL-C goal was similar (77.49%). However, the rate of escalation was much higher (48.49%), which could be attributed to hospitalization in the cardiology department. Post hoc analysis of the SANTORINI study showed that recent hospitalization for cardiovascular disease increased the likelihood of intensification of lipid-lowering therapy [6].

Our study shows that the decision about escalation was mainly based on the classical risk factor or the evident presence of atherosclerotic cardiovascular disease. Predominantly, the decision is based on proper risk estimation, and in the majority of cases, on the distance between the goal and the initial LDL-C. That is also confirmed by the SANTORINI study [6]. Unfortunately, despite growing evidence for amplifiers of cardiovascular risk, the decision-making process relies on the classical risk factors such as dyslipidemia and hypertension.

Escalation of therapy is less frequent than anticipated. Even in our study, 49.10% of patients in the no-escalation group were in the very high-risk category, and only 33.03% were within the LDL-C goal. Careful examination and evaluation of cardiovascular risk should be a crucial part of each cardiovascular hospitalization, as this has the highest longitudinal impact on adherence to therapy. Initiation of statin therapy during hospitalization for ACS was the strongest predictor of treatment continuation at 6-month follow-up [20]. Intensive hypolipidemic treatment is not only safe but also very effective in achieving target goals. In a study conducted among patients undergoing STEMI, initiation of double or triple therapy resulted in 100% of patients reaching the target LDL-C goal at 12-month follow-up [21]. Coordinated care programs including intensive monitoring and reward for reaching goals can provide additional benefit for post-MI patients [22,23,24]. The prescription of statin and ezetimibe increased with growth of cardiovascular risk. The majority of patients with high/very high/extreme risk received statins. Ezetimibe prescription at discharge was highest among patients with extreme risk (50%). In our study the recommended double and triple therapy was rarely initiated despite the risk and distance from LDL-C goal.

Other factors influencing risk category and treatment decisions have been updated in the recent ESC guidelines. A special role was given to elevated Lp (a) [>50 mg/dL (>105 nmol/L)] [4]. The recommendation to measure Lp (a) at least once per lifetime was already suggested in previous guidelines [4,5]. This unique recommendation was presented by Polish Societies in 2024, suggesting more intensive prevention and treatment among patients with elevated Lp (a) [16]. However, despite growing evidence on the relation of Lp (a) and cardiovascular disease, our study showed that it was not included in decisions regarding intensification of treatment. A study based on the US Family Heart Database showed that high-impact LDL-C reduction therapy, especially when including PCSK-9i, can neutralize the influence of Lp (a) on recurrence of atherosclerotic cardiovascular disease [25]. Thus, intensive treatment should be the standard of care among individuals who experience cardiovascular events and have elevated Lp (a) [26].

Regarding other factors which should influence the decision regarding hypolipemic treatment, in our study and in other studies, obesity and increase in BMI were associated with an increase in statin intensity [27].

Initiatives targeting screening for potential risk factors should be connected with efficient interventions, including lifestyle modification and initiation of treatment with proven benefits, to achieve cost-effective results. In Poland, two major screening programs have been started. The first includes performing a lipid profile during child assessment at the age of 6 years; the second, called “My Health”, is a national program involving a basic health evaluation for those 20 years old or more. That program includes Lp (a) testing for individuals between 20 and 40 years old [28]. However, such programs have to be connected with general educational efforts to increase awareness both among patients and physicians.

An additional factor relies on adherence to the treatment proposed by previous medical professionals before hospitalization. Low prevalence of lipid-lowering therapy at admission can be caused by withdrawal from previously initiated therapy. Interventions regarding patient motivation and knowledge regarding the disease and goals of the treatment can improve adherence [29]. Perception of the treatment’s side effects and a high number of medications decreases compliance. The factors increasing risk of non-adherence also include young patients (<50 y), females, smokers, new users of lipid-lowering medications, and having doubts about the safety of the treatment [30]. However, risk can be reduced with time spent by the physician explaining different aspects of cholesterol and cardiovascular disease relations [31]. There is also a phenomenon of overestimation of achieved treatment results, when physicians assume that a large portion of the patients are within the target goal [32]. Electronic system alerts can improve screening and treatment for dyslipidemia by general practitioners [33].

## 5. Limitations

This study has several limitations that should be considered when interpreting the results. Statistical power might be limited, and generalizability of the findings may be reduced because of relatively small sample size (*n* = 431). Observed associations provide valuable preliminary insights. Nevertheless, larger and more heterogeneous cohorts are required to validate results and to explore potential subgroup effects.

Adherence to lipid-lowering therapy and the rationale for therapy escalation might be affected by the study’s retrospective, observational design. Data were obtained from medical records, which may have been incomplete or variably documented.

Single-center analysis might have influenced the decision to escalate therapy and the achieved lipid targets. The availability of lipid-lowering agents such as ezetimibe or PCSK9 inhibitors, treatment strategies, and prescribing practices could differ across healthcare systems.

Several potentially relevant clinical and behavioral factors were not captured in this analysis, such as dietary patterns, genetic predisposition, and the patients’ socioeconomic status. Despite these limitations, the study provides important real-world findings that may help develop more individualized approaches to dyslipidemia in high-risk cardiovascular populations.

Future studies should be expanded to include other centers. The current results should inform the design of prospective studies. An important next step would be to validate a risk score model. This could help doctors identify patients most likely to require escalation of lipid-lowering therapy. Incorporating clinical, biochemical, and behavioral variables into such a tool could facilitate individualized treatment decisions and improve adherence to guideline-recommended LDL-C targets. Furthermore, prospective studies assessing the impact of early and appropriately targeted escalation of therapy on adverse cardiovascular events support the evidence for increasing active approaches to dyslipidemia management in high- and very high-risk populations.

## 6. Conclusions

The prevalence of patients within the LDL-C goal is low. The main factors influencing the decision to initiate LLT include classical risk factors or established atherosclerotic cardiovascular disease. In this specific study population, Lp (a) did not affect the escalation of therapy, despite growing interest among medical practitioners. This may reflect limited statistical power, selection of very high-risk patients, and current gaps in clinicians’ implementation of Lp (a)-guided strategies. not reaching the LDL-C goal was the strongest predictor of escalation of LLT; however, many patients not within the goal do not undergo intensification, despite high/very-high risk.

## Figures and Tables

**Table 1:** Risk modifiers for consideration beyond the risk estimation based on the SCORE2 and SCORE2-OP algorithms, or diseases with specific cardiovascular risk.

Diseases with Specific Cardiovascular Risk		
Documented atherosclerotic cardiovascular disease Markedly elevated single risk factors (dyslipidemia and hypertension) Diabetes mellitus Familial dyslipidemias Chronic kidney disease		
Risk modifiers		
2019 ESC/EAS Guidelines for the management of dyslipidaemias [5]	2021 ESC Guidelines on cardiovascular disease prevention in clinical practice [3]	2025 Focused Update of the 2019 ESC/EAS Guidelines for the management of dyslipidaemias [4]
Social deprivation: the origin of many of the causes of CVD. Obesity and central obesity as measured by the body mass index and Waist circumference, respectively. Physical inactivity. Psychosocial stress including vital exhaustion. Family history of premature CVD Chronic immune-mediated inflammatory disorder. Major psychiatric disorders. Treatment for human immunodeficiency virus infection. Atrial fibrillation. Left ventricular hypertrophy. Chronic kidney disease. Obstructive sleep apnoea syndrome. Non-alcoholic fatty liver disease. Chronic immune-mediated inflammatory diseases Severe mental illness	Psychosocial factors High-risk ethnicity Arterial stiffness Frailty Family history of premature CVD Genetics Socioeconomic determinants Environmental exposure (air pollution) Body composition Cancer Chronic obstructive pulmonary disease Inflammatory conditions Migraine Sleep disorders and Obstructive sleep apnea Mental disorders Sex-specific conditions (preeclampsia and/or pregnancy induced hypertension) Infections Atrial fibrillation Heart failure	Family history of premature CVD High-risk ethnicity Stress symptoms and psychosocial stressors Social deprivation Obesity Physical inactivity Chronic immune-mediated/inflammatory disorders Major psychiatric disorders History of premature menopause Pre-eclampsia or other hypertensive disorders of pregnancy Human immunodeficiency virus infection Obstructive sleep apnoea syndrome Persistently elevated hs-CRP (>2 mg/L) Elevated Lp (a) [>50 mg/dL (>105 nmol/L)]

**Table 2:** Cardiovascular risk factors’ influence on the escalation of lipid-lowering therapy.

Variable	Total *n* = 431	Escalation of Lipid-Lowering Therapy *n* = 209	No-Escalation of Lipid-Lowering Therapy *n* = 222	Odds Ratio	95% CI	*p* Value
Age [y.]	69.00± 19.00	68.00 ± 15.00	69.50 ± 24.00	1.007	0.995–1.020	0.969
Sex (male)	45.01% (194)	47.37% (99)	42.79% (95)	1.203	0.823–1.759	0.340
Chronic coronary syndrome	29.00% (125)	37.32% (78)	21.17% (47)	2.217	1.447–3.398	<0.001
Acute coronary syndrome	4.18% (18)	6.70% (14)	1.80% (4)	3.913	1.267–12.087	0.018
Past MI	12.99% (56)	12.92% (27)	13.06% (29)	0.987	0.563–1.732	0.964
Acute stroke	5.34% (23)	6.70% (14)	4.05% (9)	1.699	0.719–4.014	0.227
Past stroke	9.74% (42)	9.09% (19)	10.36% (23)	0.865	0.457–1.640	0.657
PAD	5.34% (23)	6.22% (13)	4.50% (10)	1.406	0.603–3.280	0.430
Carotid artery stenosis	22.04% (95)	26.32% (55)	18.02% (40)	1.625	1.026–2.575	0.039
Hypertension	75.17% (324)	80.38% (168)	70.27% (156)	1.734	1.109–2.709	0.016
Systolic blood pressure [mmHg]	138.50 ± 2.72	138.00 ± 4.15	140.00 ± 2.86	0.991	0.956–1.029	0.657
Diastolic blood pressure [mmHg]	80.00 ± 2.07	80.00 ± 2.44	79.50 ± 3.75	0.998	0.951–1.048	0.949
Dyslipidemia	74.71% (322)	82.78% (173)	67.12% (149)	2.354	1.493–3.712	<0.001
Lp (a) [mg/dL]	13.80 ± 31.70	14.90 ± 34.10	12.20 ± 27.70	1.001	0.996–1.005	0.777
Lp (a) > 50 mg/dL	21.11% (91)	21.05% (44)	21.17% (47)	0.993	0.625–1.577	0.976
Lp (a) > 180 mg/dL	1.86% (8)	1.44% (3)	2.25% (5)	0.632	0.149–2.678	0.533
TC [mg/dL]	162.00 ± 68.00	176.00 ± 69.00	145.00 ± 59.00	1.015	1.011–1.020	<0.001
LDL-C [mg/dL]	95.00 ± 61.00	112.00 ± 62.00	82.00 ± 45.00	1.021	1.015–1.027	<0.001
HDL-C [mg/dL]	51.00 ± 20.00	50.00 ± 19.00	51.00 ± 20.00	0.999	0.987–1.010	0.818
TG [mg/dL]	106.00 ± 66.00	115.00 ± 73.00	99.00 ± 58.00	1.002	1.000–1.003	0.125
Non-HDL-C [mg/dL]	108.00 ± 59.00	122.00 ± 59.00	95.50 ± 47.00	1.018	1.013–1.023	<0.001
Distance from LDL-C goal	31.00 ± 60.00	56.5 ± 63.00	12.00 ± 42.00	1.029	1.022–1.036	<0.001
Not-reached LDL-C goal	77.49% (334)	93.78% (196)	62.16% (138)	9.177	4.920–17.119	<0.001
Prediabetes	7.89% (34)	11.00% (23)	4.95% (11)	2.372	1.126–4.997	0.023
Diabetes	27.84% (120)	27.75% (58)	27.93% (62)	0.991	0.650–1.510	0.967
Glycemia	102.00 ± 2.44	103.00 ± 2.57	99.5 ± 4.01	0.999	0.996–1.003	0.758
HbA1C [%]	5.6 ± 0.28	5.7 ± 0.13	5.6 ± 0.53	0.986	0.949–1.025	0.484
Obesity	31.55% (136)	36.36% (76)	27.03% (60)	1.543	1.025–2.322	0.038
BMI	28.63 ±0.28	29.76 ± 0.44	28.29 ± 0.36	1.042	1.005–1.081	0.027
Chronic kidney disease	24.36% (105)	23.44% (49)	25.23% (56)	0.908	0.584–1.411	0.667
GFR [mL/min]	81.00 ± 1.51	81.00 ± 1.87	81.00 ± 231	0.998	0.992–1.003	0.392
Heart failure	41.53% (179)	42.58% (89)	40.54% (90)	1.088	0.741–1.596	0.667
NT-pro BNP [pg/mL]	350.70 ± 116.75	340.65 ± 163.60	359.15 ± 166.39	1.000	0.999–1.000	0.941
LVEF [%]	55.00 ± 0.87	55.00 ± 1.38	55.00 ± 1.07	0.985	0.956–1.015	0.329
Atrial fibrillation	33.87% (146)	30.14% (63)	37.39% (83)	0.7226	0.484–1.080	0.1128
Asthma	4.87% (21)	5.26% (11)	4.50% (10)	1.1778	0.490–2.833	0.7149
COPD	6.03% (26)	7.66% (16)	4.50% (10)	1.7575	0.779–3.966	0.1744
Active smoking	14.85% (64)	16.75% (35)	13.06% (29)	1.339	0.786–2.281	0.284
History of smoking	29.93% (129)	33.97% (71)	26.13% (58)	1.455	0.961–2.201	0.076
Family history of cardiovascular diseases	33.64% (145)	34.93% (73)	32.43% (72)	1.118	0.750–1.668	0.584
Age at the first episode [y.]	62.00 ± 24.00	62.00 ± 21.00	66.00 ± 26.00	0.974	0.936–1.014	0.990
Premature Cardiovascular disease (M < 55 y., F < 60 y.)	11.60% (50)	10.53% (22)	12.61% (28)	0.815	0.450–1.476	0.500

MI—Myocardial infarction; PAD—Peripheral artery disease; Lp (a)—Lipoprotein a; TC—Total Cholesterol, LDL-C—low-density lipoprotein-cholesterol; HDL-C—High-Density Lipoprotein Cholesterol; TG—Triglycerides; Non-HDL-C—Non-High-Density Lipoprotein Cholesterol.

**Table 3:** Comparison of the established cardiovascular risk (ESC 2025 [11]) and initial LDL-C goals reached between the escalation and no-escalation groups.

	Total *n* = 431	Escalation of Lipid-Lowering Therapy *n* = 209	No Escalation of Lipid-Lowering Therapy *n* = 222	*p* Value
Low cardiovascular risk	7.66% (33)	2.39% (5)	12.61% (28)	<0.001
Reached specific LDL-C goal for low risk (<116 mg/dL)	51.52% (17)	20.00% (1)	57.14% (16)	0.125
Moderate cardiovascular risk	9.05% (39)	6.22% (13)	11.71% (26)	0.047
Reached specific LDL-C goal for moderate risk (<100 mg/dL)	51.28% (20)	38.46% (5)	57.69% (15)	0.257
High cardiovascular risk	19.72% (85)	18.18% (38)	21.17% (47)	0.436
Reached specific LDL-C goal for High risk (LDL-C < 70 mg/dL)	23.53% (20)	7.89% (3)	36.17% (17)	0.002
Very high cardiovascular risk	54.29% (234)	59.81% (125)	49.10% (109)	0.026
Reached specific LDL-C goal for very high risk (<55 mg/dL)	16.67% (39)	2.40% (3)	33.03% (36)	<0.001
Extreme cardiovascular risk	9.28% (40)	12.92% (27)	5.86% (13)	0.012
Reached specific LDL-C goal for extreme risk (<40 mg/dL)	2.50% (1)	0.00% (0)	7.69% (1)	0.144

**Table 4:** Impact of the lipid profile and Lp (a) on the escalation of lipid-lowering therapy. The multivariable logistic regression was repeated for various LDL-C levels.

Variable	Odds Ratio	95% CI	*p* Value
LDL-C > 100 mg/dL			
Lp (a)	0.997	0.991–1.004	0.478
TC	1.005	0.997–1.013	0.247
LDL-C	1.007	0.997–1.017	0.180
HDL-C	0.990	0.972–1.008	0.264
TG	1.004	0.999–1.009	0.086
Non-HDL-C	1.007	0.998–1.015	0.117
LDL-C > 70 mg/dL			
Lp (a)	1.001	0.995–1.006	0.769
TC	1.011	1.005–1.016	<0.001
LDL-C	1.015	1.008–1.022	<0.001
HDL-C	0.989	0.976–1.003	0.125
TG	1.005	1.001–1.0091	0.008
Non-HDL-C	1.014	1.007–1.021	<0.001
LDL-C > 55 mg/dL			
Lp (a)	1.001	0.996–1.006	0.689
TC	1.011	1.006–1.016	<0.001
LDL-C	1.016	1.009–1.022	<0.001
HDL-C	0.991	0.979–1.003	0.156
TG	1.001	0.999–1.003	0.212
Non-HDL-C	1.014	1.008–1.019	<0.001
Not within target LDL-C goal			
Lp (a)	1.000	0.995–1.005	0.951
TC	1.011	1.005–1.016	<0.001
LDL-C	1.014	1.009–1.021	<0.001
HDL-C	0.980	0.938–1.024	0.359
TG	1.001	0.999–1.002	0.399
Non-HDL-C	1.012	1.007–1.018	<0.001

**Table 5:** Comparison of pharmacotherapy between initial treatment at admission and discharge treatment.

Pharmacotherapy	Initial Treatment	Discharge Treatment	*p* Value
Statin	45.94% (198)	74.71% (322)	<0.001
Simvastatin	1.86% (8)	0.23% (1)	0.019
Pitavastatin	0.70% (3)	2.09% (9)	0.081
Rosuvastatin	23.20% (100)	40.60% (175)	<0.001
Atorvastatin	20.19% (87)	31.79% (137)	<0.001
Low-to-moderate intensity statin	26.68% (115)	32.95% (142)	0.044
Simvastatin 20 mg	1.39% (6)	0.23% (1)	0.058
Simvastatin 40 mg	0.46% (2)	0.00% (0)	0.157
Pitavastatin 2 mg	0.46% (2)	1.62% (7)	0.094
Pitavastatin 4 mg	0.23% (1)	0.46% (2)	0.563
Rosuvastatin 5–15 mg	9.28% (40)	12.06% (52)	0.185
Atorvastatin 10–30 mg	14.85% (64)	18.56% (80)	0.144
High-intensity statin	19.26% (83)	41.76% (180)	<0.001
Rosuvastatin 20–40 mg	13.92% (60)	28.54% (123)	<0.001
Atorvastatin 40–80 mg	5.34% (23)	13.23% (57)	<0.001
Ezetimibe	7.89% (34)	14.15% (61)	0.003
Ezetimibe and statin	6.26% (27)	12.76% (55)	0.001
PCSK-9i	0.46% (2)	1.39% (6)	0.155
Statin, ezetimibe, and PCSK-9i)	0.23% (1)	0.93% (4)	0.178
Fibrate	2.78% (12)	3.48% (15)	0.558

**Table 6:** Comparison of pharmacotherapy at the admission between patients within and not-within LDL-C goal.

Pharmacotherapy	Within LDL-C Goal *n* = 97	Not within LDL-C Goal *n* = 334	*p* Value
Statin	62.89% (61)	41.02% (137)	<0.001
Simvastatin	1.03% (1)	2.10% (7)	0.494
Pitavastatin	0.00% (0)	0.90% (3)	0.349
Rosuvastatin	35.05% (34)	19.76% (66)	0.002
Atorvastatin	26.80% (26)	18.26% (61)	0.065
Low-to-moderate intensity statin	36.08% (35)	23.95% (80)	0.017
Simvastatin 20 mg	0.00% (0)	1.80% (6)	0.183
Simvastatin 40 mg	1.03% (1)	0.30% (1)	0.351
Pitavastatin 2 mg	0.00% (0)	0.60% (2)	0.445
Pitavastatin 4 mg	0.00% (0)	0.30% (1)	0.590
Rosuvastatin 5–15 mg	14.43% (14)	7.78% (26)	0.047
Atorvastatin 10–30 mg	20.62% (20)	13.17% (44)	0.069
High-intensity statin	26.80% (26)	17.07% (57)	0.032
Rosuvastatin 20–40 mg	20.62% (20)	11.98% (40)	0.030
Atorvastatin 40–80 mg	6.19% (6)	5.09% (17)	0.672
Ezetimibe	13.40% (13)	6.29% (21)	0.022
Ezetimibe and statin	12.37% (12)	4.49% (15)	0.005
PCSK-9i	1.03% (1)	0.30% (1)	0.351
Statin, ezetimibe, and PCSK-9i)	1.03% (1)	4.49% (0)	0.063
Fibrate	2.06% (2)	2.99% (10)	0.623

**Table 7:** Comparison of pharmacotherapy at discharge with subdivision regarding cardiovascular risk category.

Pharmacotherapy	Low Risk *n* = 33	Moderate Risk *n* = 39	High Risk *n* = 85	Very High Risk *n* = 234	Extreme Risk *n* = 40	*p* Value
Statin	21.21% (7)	48.72% (19)	76.47% (65)	83.33% (195)	90.00% (36)	<0.001
Simvastatin	0.00% (0)	0.00% (0)	0.00% (0)	0.43% (1)	0.00% (0)	0.932
Pitavastatin	0.00% (0)	5.13% (2)	0.00% (0)	2.14% (5)	5.00% (2)	0.204
Rosuvastatin	9.09% (3)	23.08% (9)	35.29% (30)	47.01% (110)	57.50% (23)	<0.001
Atorvastatin	12.12% (4)	20.51% (8)	41.18% (35)	33.76% (79)	27.50% (11)	0.015
Low-to-moderate intensity statin	18.18% (6)	33.33% (13)	40.00% (34)	32.91% (77)	30.00% (12)	0.255
Simvastatin 20 mg	0.00% (0)	0.00% (0)	0.00% (0)	0.43% (1)	0.00% (0)	0.932
Simvastatin 40 mg	0.00% (0)	0.00% (0)	0.00% (0)	0.00% (0)	0.00% (0)	1.000
Pitavastatin 2 mg	0.00% (0)	5.13% (2)	0.00% (0)	1.28% (3)	5.00% (2)	0.093
Pitavastatin 4 mg	0.00% (0)	0.00% (0)	0.00% (0)	0.85% (2)	0.00% (0)	0.792
Rosuvastatin 5–15 mg	6.06% (2)	10.26% (4)	15.29% (13)	12.39% (29)	10.00% (4)	0.688
Atorvastatin 10–30 mg	12.12% (4)	17.95% (7)	24.71% (21)	17.95% (42)	15.00% (6)	0.488
High-intensity statin	3.03% (1)	15.38% (6)	36.47% (31)	50.43% (118)	60.00% (24)	<0.001
Rosuvastatin 20–40 mg	3.03% (1)	12.82% (5)	20.00% (17)	34.62% (81)	47.50% (19)	<0.001
Atorvastatin 40–80 mg	0.00% (0)	2.56% (1)	16.47% (14)	15.81% (37)	12.50% (5)	0.026
Ezetimibe	0.00% (0)	2.56% (1)	5.88% (5)	14.96% (35)	50.00% (20)	<0.001
Ezetimibe and statin	0.00% (0)	2.56% (1)	4.71% (4)	14.53% (34)	40.00% (16)	<0.001
PCSK-9i	0.00% (0)	0.00% (0)	0.00% (0)	0.85% (2)	10.00% (4)	<0.001
Triple therapy (statin, ezetimibe, and PCSK-9i)	0.00% (0)	0.00% (0)	0.00% (0)	0.43% (1)	7.50% (3)	<0.001
Fibrate	3.03% (1)	2.56% (1)	4.71% (4)	2.99% (7)	2.50% (1)	0.944

**Table 8:** The types of escalation of lipid-lowering therapy with subdivision regarding the distance to LDL-C goal.

Escalation of Lipid-Lowering Therapy	Total 48.49% *n* = 209	Distance to LDL-C Goal < 50 mg/dL *n* = 92	Distance to LDL-C Goal ≥ 50 mg/dL *n* = 117	*p* Value
Statin initiation	39.71% (83)	53.26% (49)	29.06% (34)	<0.001
Dose increasing	26.32% (55)	38.04% (35)	17.09% (20)	<0.001
Low-to-moderate into high intensity statin	2.87% (6)	2.17% (2)	3.42% (4)	0.593
Ezetimibe initiation	12.92% (27)	9.78% (9)	15.38% (18)	0.231
Up-front, combined lipid therapy (statin and ezetimibe)	5.26% (11)	1.09% (1)	8.55% (10)	0.017
PCSK-9i initiation	1.91% (4)	0.00% (0)	3.42% (4)	0.073
Fibrate initiation	1.91% (4)	1.09% (1)	2.56% (3)	0.439

## Data Availability

The data presented in this study are available on request from the corresponding author.
